# Psychosocial Factors and Chronic Illness as Predictors for Anxiety and Depression in Adolescence

**DOI:** 10.3389/fpsyg.2020.568941

**Published:** 2020-09-18

**Authors:** Laura Lacomba-Trejo, Selene Valero-Moreno, Inmaculada Montoya-Castilla, Marián Pérez-Marín

**Affiliations:** Department of Personality, Assessment and Psychological Treatments, Faculty of Psychology, University of Valencia, Valencia, Spain

**Keywords:** adolescent, chronic illness, qualitative comparative analysis, structural equation models, adjustment

## Abstract

Adolescence is a challenging time when emotional difficulties often arise. Self-esteem, good relationships with peers, and emotional competences can buffer the effects of these difficulties. The difficulties can be even greater when coupled with the presence of a chronic physical illness (CD). Our goal is to analyze psychosocial factors and CD as predictors for anxiety and depression. It was compared the results of structural equation models (SEM) with models based on qualitative comparative analysis (QCA) to analyze the possible influence of these variables on levels of anxiety-depression in adolescents with and without CD. The sample consisted of 681 adolescents, between 12 and 16 years old (*M* = 13.94, *SD* = 1.32). 61.50% were girls and 13.40% (*n* = 222) presented a CD (mainly pneumo-allergic and endocrine). They were evaluated by the Hospital Anxiety and Depression Scale, the Self-esteem Questionnaire, the Emotional Competences Questionnaire and the Strengths and Difficulties Questionnaire. The results obtained by SEM show that low self-esteem, problems with peers and low emotional competencies predict anxiety in 41% of the variance and depression in 72%. The results obtained by QCA show that the different combinations of these variables explain between 24 and 61% of low levels of anxiety and depression and 47–55% of high levels. Our data show how the presence of a CD, low self-esteem, problems with peers and problems in emotional skills play a fundamental role in explaining levels of anxiety and depression. These aspects will help provide increased resources for emotional adjustment in the educational context, facilitating the transitions to be made by adolescents.

## Introduction

Adolescence is defined as the stage of development involved in the transition between childhood and adulthood, characterized by a wide variety of changes at the biological, psychological and social changes (Andrews et al., 2020), and it is often a time of considerable personal challenges and difficulties, especially regarding mental health. According to the World Health Organization (WHO), mental health is “a state of well-being in which the individual realizes his or her own abilities, can cope with the normal stresses of life, can work productively and fruitfully, and is able to make a contribution to his or her community. The WHO points out that half of all mental disorders begin before the age of 14 (World Health Organization [WHO], 2011). Affecting mental health, anxiety and mood problems are the most common at this stage, and there is a great deal of comorbidity between these disorders (Sowislo and Orth, 2013). As a result, between 13.3 and 16.4% of children and adolescents in Spain suffer from anxiety, and between 5 and 13% suffer from depression (Del Barrio, 2015). Psychological problems in adolescence are the main cause of morbidity and disability during this period, suicide being the third most common cause of mortality among adolescents (World Health Organization [WHO], 2018). In addition, having psychological problems in childhood is associated with an increased likelihood of suffering from it in adulthood (Navarro-Pardo et al., 2012; Gaete, 2015; Duinhof et al., 2020).

As well as having psychological problems, the presence of a chronic physical illness (CD) during adolescence adds even more complexity and difficulties to the already challenging tasks associated with this stage of transition to adulthood (Extremera et al., 2018; Kim et al., 2020). The most common CD in adolescence are pneumo-allergic and endocrine diseases (Chueca et al., 2008; Alith et al., 2015). Living with a CD can significantly limit the day-to-day life of adolescents, given the changes it involves in their lifestyle (diet, exercise and medical treatment), and the need for continuous monitoring by health services (Lacomba-Trejo et al., 2018).

The CD can have an impact on the adolescent’s emotional health, social and family relationships, and academic performance (e.g., due to school absences for medical check-ups) (Valero-Moreno et al., 2020). Studies therefore indicate a higher frequency of emotional and relational difficulties in these adolescents (Cobham et al., 2020; Kim et al., 2020; Nabors, 2020), but these results deserve to be discussed, since some studies showed that the adjustment to the disease could also be more related to personal and family variables than to the disease itself (Lohan et al., 2015). Therefore, having a CD is considered a relevant factor in adjustment in adolescence, but other risk and protective factors must also be taken into account to achieve a greater understanding of adjustment (Stanton and Revenson, 2007), such as self-esteem, social support or emotional skills.

The literature has highlighted how adequate self-esteem and social support can buffer the emotional distress associated with the CD (Igai, 2020). Similarly, it has been pointed out how emotional regulation skills can facilitate the adjustment of adolescents with CD (Trindade et al., 2017; Finlay-Jones et al., 2020) psychosocial risk and protective variables are critical to the medical and psychological adjustment of adolescents with CD, as emotional or personal difficulties can impact the control of CD, worsening the course and outcome of the disease (Kenowitz et al., 2020; Valero-Moreno et al., 2020).

Our study aims to analyze the possible influence of self-esteem, peer problems and emotional competencies on anxiety-depressive clinical levels in adolescents with and without CD. For it, the results of two statistical methodologies have been compared [structural equation models (SEM) vs. models based on comparative qualitative analysis (QCA)] to analyze. As hypotheses: (H1) self-esteem and emotional competencies will be negatively related to emotional adjustment (higher levels of anxiety and depression); (H2) problems with peers will be positively related to anxiety and depression); (H3) adolescents with CD will show lower levels of self-esteem and emotional competencies, and higher levels of anxiety, depression and problems with peers.

## Materials and Methods

### Participants and Procedure

This study involved 681 adolescents from the Valencian Community. The average age of all the subjects surveyed was 13.94 years old (*SD* = 1.32), with a range of 12–16 years. The percentage of boys surveyed was 38.2%, while for girls it was 61.5%, 0.2% gender queer. 32.6% presented chronic physical diseases (CD), which were mainly pneumoallergic (asthma, allergy, atopic dermatitis) with a percentage of 27.3%, endocrine or gastrointestinal [DM1 (0.6%), celiac disease (0.6%) or obesity problems (0.3%)]. The assessment of all adolescents (with and without CD) was conducted at the same time and in a common setting, the school context. This fact facilitates better that the responses of all adolescents were more similar, and responses of adolescents with CD were less biased if there were performed within the hospital context.

The study was approved by the Bioethics Committee of the Government of the Valencian Community (CN00A/2020/42/S) and the University of Valencia’s (UV-INV_ETICA-1226194). All participants received detailed information about the aims and procedures and were informed of confidentiality. Data collection and data analysis took place between October 2019 and February 2020.

### Statistical Analysis

First, the descriptive analyses of the participants were estimated, and then calibration values for fsQCA were calculated. A structural equation model (SEM) and a fuzzy-set qualitative comparative analysis (fsQCA) were subsequently performed. In the structural equation model, (SEM) the estimate provided by the robust method of maximum likelihood estimation (ML), recommended to correct the possible absence of multivariate normality, was applied in all cases. A structural equation model was made to predict anxiety and depression (dependent variables) through emotional competencies, self-esteem, and problems with peers (independent variables). In addition, a multigroup was conducted to test the moderating effect of CD.

When performing the fuzzy-set qualitative comparative analysis, the raw data from the participants’ responses were transformed into fuzzy-set responses. First, all the missing data were deleted as suggested in the literature, and all constructs (variables) were calculated by multiplying their item scores (Villanueva et al., 2017; Giménez-Espert and Prado-Gascó, 2018; Navarro-Mateu et al., 2020). Before performing the analysis, the values were recalibrated between 0 and 1. When considering only two values as in the variable for the presence of CD, we used 0 (healthy) and 1 (CD). However, when performing the recalibration with more than two values, we had to consider the following three thresholds: the first (0) considers that an observation with this value is fully outside the set (low agreement); the second (0.5) considers a median point, neither inside nor outside the set (intermediate level of agreement); and the last value (1) considers the observation to be fully in the set (high level of agreement). With continuous variables or with factors from a survey, these three values must be introduced for an automatic recalibration of values between 0 and 1. In these cases, the literature suggests that with continuous variables or with factors, the three thresholds must be percentiles 10, 50, and 90 (Woodside, 2013). The fsQCA 2.5 software by Claude and Christopher (2014) recalibrated the values of self-esteem, peer problems, emotional intelligence and anxiety and depression (Woodside, 2013). SPSS (*Statistical Package for the Social Sciences*, version 23, ^©^IBM) was used to perform the descriptive analysis and produce the calibration values and mean differences, Cohen’s *d*, EQS (*Structural Equation Modeling Software*, version 6.3, Bentler, 1985–2016, Multivariate Software Inc.), to evaluate the psychometric properties of the instruments and structural equation models, and fsQCA software (*fuzzy qualitative comparative analysis*, version 2.5, ^©^Raging and David, 1999–2008), (Claude and Christopher, 2014) was used to perform fsQCA.

### Measures

The version of the Rosenberg Self-Esteem Scale (RSE) (Rosenberg, 1965) adapted to the Spanish context (Atienza et al., 2000) consists of 10 items [a Likert format, ranging from 1 (Strongly disagree) to 4 (Strongly agree)], and focuses on feelings of respect for and acceptance of oneself. The original study (Rosenberg, 1965) obtained an internal consistency of 0.92. The alpha scores in Spanish studies of adolescents have a reliability of 0.86 (Oliva et al., 2011). The total score ranges from 10 to 40 points, distinguishing between low (scores less than or equal to 29) and high (equal to or greater than 30) self-esteem. The psychometric properties in this study seem to be adequate: χ^2^(df) = 217.50(35); S-B χ^2^(df) = 198.73(35); S-B χ 2/df (5.68; CFI = 0.92; IFI = 0.92; RMSEA = 0.08 (IC = 0.07–0.09); α = 0.80.

The Hospital Anxiety and Depression Scale (HADS) (Zigmond and Snaith, 1983) was developed as a screening instrument for identifying non-psychiatric patients with affective disorders attending hospitals (De Las Cuevas et al., 1995). It is divided into two dimensions: the anxiety subscale (HADS-A) and the depression subscale (HADS-D). An overall score for emotional distress is obtained by adding the anxiety and depression scales. The questionnaire has been used in adolescents and young people (Saez-Flores et al., 2018) (from 10 to 23 years old), and validated for pediatric samples (Valero-Moreno et al., 2019) obtaining adequate psychometric properties (Mihalca and Pilecka, 2015; Valero-Moreno et al., 2019). Scores between 0 and 6 represent no anxiety, 7–9 possible anxiety, and scores of over 10 probable anxiety. In depression, 0–5.4 represents no depression, 5.5–7.5 possible depression, over 7.5 probable depression and for emotional distress, below 15.5 is no emotional distress, and over 15.5 emotional is probable distress (Valero-Moreno et al., 2019). The psychometric properties in the present study seem to be adequate: χ^2^(df) = 98.76(40); S-Bχ^2^(df) = 81.21(40); S-Bχ^2^/df = 2.03; CFI = 0.97; IFI = 0.97; RMSEA = 0.04 (IC = 0.03–0.05); α = 0.78 for anxiety, α = 0.68 for depression and α = 0.82 for emotional distress.

The Emotional Skills and Competence Questionnaire (ESCQ-21) is a self-report measure created by Takšić et al. (2009) with the aim of assessing emotional competence. The reduced version (ESCQ-21), which was adapted and validated in a Spanish sample (Schoeps et al., 2019) was used in this study, scored on a 6-point Likert scale, from 1 (“never”) to 6 (“always”). The scale has three different subscales: “Perception and understanding,” “Expressing and labeling” and “Handling and regulation.” It has proved to be a reliable and valid measure in several contexts, showing adequate psychometric properties (Schoeps et al., 2019). The psychometric properties in this seem to be adequate: χ^2^(df) = 573.31(186); S-Bχ^2^(df) = 447.15(186); S-Bχ^2^/df = 2.40; CFI = 0.93; IFI = 0.93; RMSEA = 0.05 (IC = 0.04–0.05); α = 0.81 for perception, α = 0.90 for expression and α = 0.78 for emotional regulation.

The Strengths and Difficulties Questionnaire (SDQ) was designed by Goodman (1997). It is a self-report measure used to assess emotional and behavioral problems related to mental health in children and adolescents (Ortuño-Sierra et al., 2016). The questionnaire consists of 25 items with a Likert format of 3 response options (from “0 = not true” to “2 = true”), which are grouped into 5 dimensions: “Emotional Symptoms,” “Behavior Problems,” “Hyperactivity”), “Problems with Peers,” and “Prosocial Behavior.” The SDQ has acceptable reliability, as well as internal, convergent, and predictive validity (Goodman et al., 1998; Ortuño-Sierra et al., 2015). Only the Peer Problems scale was used in our research. The psychometric properties in this study do not seem be adequate: α = 0.52 peer problems but for its full scale (difficulties) it was 0.71.

## Results

First, the main descriptors and calibration values for the variables studied are presented (Table 1).

**TABLE 1:** Main descriptions and calibration values.

	**Self-esteem**	**Anxiety**	**Depression**	**Peer problems**	**Emotional perception**	**Emotional expression**	**Emotional regulation**
*M*	147322.63	274.54	28.97	21.26	61115.97	66087.38	62029.35
*SD*	218434.91	510.80	75.50	23.75	64006.52	79979.84	68212.70
Min.	0	1	1	2	2	2	1
Max	1,368,576	4,096	1,024	243	279,936	279,936	279,936
**Calibration values**							
P10	1,670	8	2	6	3,840	576	2,160
P50	52,488	72	8	12	40,000	32,000	36,432
P90	442,368	787	64	37	135,000	194,400	157,464

*M, mean; SD, standard deviation; min, minimum; max, maximum; P10, 10th percentile; P50, 50th percentile; P90, 90th percentile.*

### Descriptive Analysis and Mean Difference

In the total sample of adolescents, 48.3% presented low *self-esteem*. In the subsample of healthy adolescents, 46.4% showed low self-esteem and 52.3% in the subsample of adolescents with CD. Differences in self-esteem were found between healthy people and CD (*t*_673_ = 2.48; *p* = 0.01; *d* = 0.20), with lower levels of self-esteem shown by adolescents with CD.

Related to the *problems with peers* variable: (a) in the total sample, 12.5% showed borderline scores in problems with peers, and 3.3% abnormal ones; (b) in the subsample of healthy adolescents, 11.6% showed borderline scores and 2.9% abnormal ones; (c) in the subsample of adolescents with CD, 14.4% showed borderline scores and 4.2% abnormal ones. However, no significant differences were found between healthy and CD adolescents (*t*_669_ = −0.51; *p* = 0.63; *d* = 0.05).

Meanwhile, in *emotional competencies*, the mean scores were found to be close to 4.5 in all competencies and in the two-subsample types, indicating moderate scores. No significant differences were found between the two groups (healthy vs. CD) in any emotional competence: emotional perception (*t*_676_ = −0.73; *p* = 0.47; *d* = 0.05), emotional expression (*t*_678_ = 0.63; *p* = 0.53; *d* = 0.05) and emotional regulation (*t*_674_ = −0.69; *p* = 0.49; *d* = 0.06).

Regarding *anxiety*: (a) 57.9% of the total sample showed anxious symptoms and 27.8% of these presented an anxiety disorder requiring psychological treatment; (b) in the subsample of healthy adolescents, 52.4% showed anxious symptoms (22.4% of these implied an anxiety disorder that would need psychological treatment; (c) in the sample of adolescents with CD, 69.2% showed anxious symptoms (and psychological treatment was recommended in 38.9% of these).

For *depression*: (a) in the total sample, 21.8% showed depressive symptoms (8.7% of these being a possible psychological disorder); (b) in the healthy sample, 19.5% had symptoms, and 6.8% of these a possible disorder; (c) in the adolescents with CD, 26.8% showed symptoms, 12.7% of these had a possible disorder requiring psychological treatment.

Finally, differences were found between the two groups for *anxiety and depression*, with adolescents with CD presenting higher levels of anxiety (*t*_676_ = −2.96; *p* ≤ 0.01; *d* = 0.42) and depression (*t*_675_ = −5.21; *p* ≤ 0.001; *d* = 0.25).

### Structural Equation Model (SEM)

The results of the causal relationships model showed a good overall fit: χ^2^ = 2361.66, df = 1012, *p* ≤ 0.001; S-Bχ^2^ = 1964.73, df = 1012, *p* ≤ 0.001; S-BX^2^/df = 1.94; RMSEA = 0.037 (IC = 0.035–0.040); CFI = 0.90; IFI = 0.90.

**FIGURE 1:**
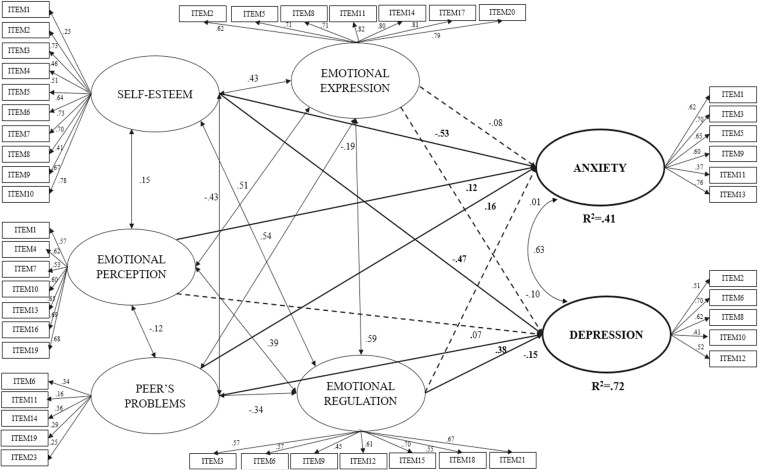
SEM model between the dimensions of emotional intelligence, self-esteem, peer problems predicting anxiety and depression. The continuous lines indicate correlation relationships (two arrows), prediction relationships (single arrowhead) and the dotted lines indicate non-significant relationships. χ^2^ = 2361.66, df = 1012, *p* ≤ 0.001; S-Bχ^2^ = 1964.73, df = 1012, *p* ≤ 0.001; S-BX^2^/df = 1.94; RMSEA = 0.037 (IC = 0.035–0.040); CFI = 0.90; IFI = 0.90.

Figure 1 shows the standardized coefficients of each of the relationships that have proven to be statistically significant predictors of the anxiety and depression. The model explained 41% (*R*^2^ = 0.41) of the variance of *anxiety* and the factors of self-esteem, peer’s problems and emotional perception were found to present a statistically significant positive relationship for peer problems (β = 0.16) and emotional perception (β = 0.12) and a negative relationship with self-esteem (β = −0.53), respectively. The model explained 72% (*R*^2^ = 0.72) of the variance of *depression* and it presented a statistically significant positive relationship for peer problems (β = 0.38) and a negative relationship negative with self-esteem (β = −0.47) and emotional regulation (β = −0.15), respectively. All the independent variables were found to be interrelated, and self-esteem was positively related to emotional competencies and negatively related to peer problems. This latter variable was also negatively related to emotional competencies. The dependent variables were also positively correlated with each other (*r* = 0.63). The moderating effect of the structural equation model could not be determined because the indices for the disease sample did not show a goodness of fit [χ^2^ = 1676.74 df = 1012, *p* ≤ 0.001; S-Bχ^2^ = 1455.99, df = 1012, *p* ≤ 0.001; S-BX^2^/df = 1.44; RMSEA = 0.045 (IC = 0.039–0.049); CFI = 0.87; IFI = 0.87].

### Comparative Qualitative Analysis of Fuzzy Sets (fsQCA)

#### Necessary Analysis

Based on the results obtained, in the necessary analysis there appears to be no necessary condition for the occurrence or non-occurrence of anxiety or depression, as all the consistency values were under 0.90 (Ragin, 2008; Table 2).

**TABLE 2:** Necessary analysis for anxiety and depression.

	**High levels of anxiety**		**Low levels of anxiety**		**High levels of depression**		**Low levels of depression**	
	**Cons**	**Cov**	**Cons**	**Cov**	**Cons**	**Cov**	**Cons**	**Cov**
Disease	0.39	0.54	0.27	0.47	0.36	0.48	0.29	0.52
Healthy	0.61	0.40	0.73	0.60	0.64	0.41	0.71	0.59
High self-esteem	0.45	0.45	0.65	0.81	0.41	0.41	0.66	0.84
Low self-esteem	0.81	0.65	0.56	0.56	0.84	0.66	0.53	0.54
High peer problems	0.68	0.60	0.56	0.63	0.69	0.60	0.54	0.61
Low peer problems	0.58	0.51	0.65	0.72	0.55	0.48	0.65	0.73
High emotional perception	0.82	0.54	0.78	0.66	0.78	0.51	0.80	0.68
Low emotional perception	0.59	0.64	0.46	0.76	0.51	0.66	0.43	0.72
High emotional expression	0.52	0.51	0.60	0.74	0.48	0.46	0.63	0.77
Low emotional expression	0.74	0.60	0.60	0.61	0.77	0.61	0.57	0.59
High emotional regulation	0.52	0.50	0.61	0.74	0.48	0.44	0.65	0.78
Low emotional regulation	0.72	0.60	0.58	0.61	0.77	0.63	0.54	0.57

*Cons, consistency; Cov, coverage; Condition needed, consistency = 0.90.*

#### Sufficiency Analysis

The combination of conditions that led to high and low levels of anxiety and depression (Table 3) were calculated in the sufficiency analyses. This is based on the premise that in fsQCA a model is informative when the consistency is around or above 0.74 (Eng and Woodside, 2012).

**TABLE 3:** Summary of the main sufficient conditions for the intermediate solution of anxiety and depression.

**Frequency cut-off 1**	**High levels of anxiety Consistency Cutoff:0.85**			**Low levels of anxiety Consistency Cutoff:0.92**			**High levels of depression Consistency Cutoff:0.81**			**Low levels of depression Consistency Cutoff:0.91**		
	**1**	**2**	**3**	**1**	**2**	**3**	**1**	**2**	**3**	**1**	**2**	**3**
Disease	•	•	•	°	°			•	•		°	°
Self-esteem	°	°		•	•	•	°	°	°	•	•	•
Peer problems	•		•		°	°	•		•			°
Emotional perception						°				•		
Emotional expression	°	•	•	°			°	°				
Emotional regulation		°	°		°	•	°	°	°	•	•	
Raw coverage	0.19	0.12	0.11	0.27	0.21	0.20	0.44	0.22	0.19	0.46	0.35	0.34
Unique coverage	0.09	0.02	0.01	0.04	0.02	0.06	0.28	0.03	0.02	0.02	0.01	0.08
Consistency	0.83	0.86	0.87	0.87	0.94	0.94	0.83	0.81	0.85	0.91	0.90	0.91
Total consistency			**0.82**			**0.88**			**0.78**			**0.88**
Total coverage			**0.24**			0.47			**0.55**			**0.61**

*•, presence of condition; °, absence of condition; ∼, absence of condition. Expected vector for anxiety and depression: 1.0.1.0.0.0 (0: absent; 1: present). Expected vector for ∼ anxiety and depression: 0.1.0.1.1.1 using the format of Fiss (2011).*

The intermediate solution indicated four combinations of causal conditions that can produce high levels of *anxiety* which accounted for 24% of cases (Overall Consistency = 0.82; Overall Coverage = 0.24) and six combinations of causal conditions that lead to low levels of anxiety that explained 47% of the cases (Overall Consistency = 0.88; Overall Coverage = 0.47) (Table 3). In the prediction of high levels of anxiety, the most relevant pathway for predicting high anxiety was the result of the interaction of low emotional expression, high levels of peer problems, low self-esteem and having a CD (Raw coverage = 0.19; Consistency = 0.86). Meanwhile, in the prediction of low levels of anxiety, the most relevant combination for predicting low anxiety was the result of the interaction of low emotional expression, high self-esteem and being healthy (Raw coverage = 0.27; Consistency = 0.87).

For *depression*, the intermediate solution indicated seven combinations of causal conditions that can lead to high levels of depression, which accounted for 55% of cases (Overall Consistency = 0.78; Overall Coverage = 0.55), and five pathways leading to low levels of depression that explained 61% of the cases (Overall Consistency = 0.89; Overall Coverage = 0.61) (Table 3). The most relevant combination for predicting high depression was the result of the interaction of low emotional regulation and emotional expression, high levels of peer problems and low self-esteem (Raw coverage = 0.44; Consistency = 0.83). Meanwhile, the most relevant pathway for predicting low depression was the result of the combination of high emotional regulation and emotional expression and high self-esteem (Raw coverage = 0.46; Consistency = 0.91).

## Discussion

It is necessary to assess the adjustment of adolescents in the school context, considering important variables as their emotional competencies, self-esteem, relationships with their peers and emotional difficulties. This aspect is even more crucial when we consider the presence of a CD during this period. Various studies (Chen et al., 2020; Duinhof et al., 2020; Wang et al., 2020) evaluate the impact of these variables on emotional adjustment in a linear fashion. However, there are virtually no studies that address these issues by comparing SEM and QCA methodologies, and those involving a comparative study of these variables considering the presence or absence of a CD in adolescence are even scarcer.

Regarding the objective of studying the psychological repercussions of CD in adolescence we would like to highlight the areas discussed below. We could do this by comparing the results of SEM models with QCA models to analyze the possible influence of self-esteem, peer problems, and emotional competencies on levels of anxiety-depression in adolescents with and without CD.

For H1 and H2, the results obtained with SEM models are generally consistent with previous studies (Martínez-Monteagudo et al., 2019; Schoeps et al., 2019; Chen et al., 2020; Duinhof et al., 2020). Adolescents who show high levels of anxiety also have low self-esteem, greater problems with their peers, and greater emotional perception. These findings are consistent with other studies, which indicated that levels of emotional perception or attention should be intermediate to be considered healthy (Navarro-Mateu et al., 2020). Similarly, adolescents with more depressive symptoms present lower self-esteem and worse emotional regulation, as well as more problems with their peers. SEM model highlights the close relationship between anxious and depressive symptoms, as already observed in previous studies (Sowislo and Orth, 2013). It has observed how lower self-esteem is associated with more peer problems and poorer emotional skills (Anto and Jayan, 2016; Wang et al., 2020). Self-esteem in both cases (anxiety and depression) is the variable that best predicts the presence of emotional symptoms, and the one that is most closely associated with emotional skills. The literature has pointed out how deficits in self-esteem and emotional skills/competencies are risk factors in the development and maintenance of psychological problems (Martínez-Monteagudo et al., 2019; Schoeps et al., 2019; Wang et al., 2020). If problems with self-esteem and emotional skills are accompanied by peer problems, the impact on psychological problems may be even greater, since less social support is considered a risk factor for physical-emotional health (Vicent et al., 2017; Chen et al., 2020).

Meanwhile, H3 is partially accepted since, at a descriptive level, adolescents with CD show a higher level of anxious and depressive symptoms, lower self-esteem, and more peer problems. However, the moderating role in the SEM model could not be verified.

In response to the comparison between the two data analysis methodologies, most studies in the health sphere have focused on analyzing the levels of anxious and depressive symptoms using linear models (Cobham et al., 2020; Duinhof et al., 2020; Kim et al., 2020; Navarro-Pardo et al., 2012) such as SEM, but they have barely studied the aspects related to this symptomatology using other non-linear relationship analyses, and examined the combination of two complementary methodologies such as SEM and QCA. This combination of methodologies gives us the opportunity to study the relationship between the variables analyzed in much more depth. The QCA models allow us to incorporate the observation of the different paths or combinations that lead to the same result (equifinality).

Our results suggest that although none of the conditions are necessary for anxious symptoms, adolescents with a CD who had low self-esteem, problems with their peers and difficulties in emotional expression and regulation presented high levels of anxiety. Similarly, the teens who did not have a CD, but had high levels of self-esteem and enjoyed positive relationships with their peers showed low levels of anxiety (even when they presented difficulties in emotional skills). Our research therefore points to the impact of CD on the lives of adolescents (Cobham et al., 2020; Kim et al., 2020; Nabors, 2020), as well as the importance of a strong social support group and good self-esteem (Valero-Moreno et al., 2020).

There are also no necessary variables for depressive symptoms, but we observed that high levels of clinical depression were associated with the combination of presence of a CD, low self-esteem, peer problems, low expression, and emotional regulation. Similarly, low levels of depression were associated with the combination of absence of CD, high self-esteem, good relationships with peers and a good ability to regulate emotions. Our results are consistent with previous studies which suggested that high self-esteem, good social support and adequate emotional skills act as an important protector against psychopathology (Schoeps et al., 2019; Valero-Moreno et al., 2020). Based on the comparison of the two methodologies, it can be concluded that these variables are predictors of anxious and depressive symptoms. This result appears in both methodologies, but in the QCA models, it can be highlighted how the presence of a CD is a relevant variable in the prediction of adolescents with high and low levels of anxiety and depression. However, more studies are needed to further explore these variables to determine the role of CD in adolescent adjustment. Our data seem to indicate that it is the psychosocial variables that determine this adjustment, since the presence of CD is only seen as a variable that worsens anxiety and depression when it occurs with other risk factors such as low self-esteem, poor emotional skills or inadequate social support.

After comparing both methodologies, we observed how QCAs also allow us to account for non-linear relationships by not focusing on the individual contribution or importance of each variable. The combination of both methodologies therefore allows our work to provide a much more complete picture of the behavior of the variables (Navarro-Mateu et al., 2020).

Despite the important contributions of this study, our research has some limitations. In particular, the cross-sectional design and the selection of the sample, complicate the generalization of the data to the population. Future studies will need to increase the number of participants on a randomized basis. All the adolescents go to schools in the Valencian Community. However, state schools and private schools partially subsidized by the government in both urban and rural areas were included, thereby making the sample more representative. In addition, we have more adolescents with no health conditions in our study than those with them. However, our data reflect the reality of the Spanish schools, with fewer students with chronic conditions than students without them. On the other hand, it should be noted that the subscale of difficulties with peers obtained an internal consistency lower than recommended. This may be frequent in subcategories composed of less than 6 items (Lloret et al., 2014).

Our results promote social awareness of the importance of protective variables such as self-esteem, emotional skills, and peer group social support in adolescence. They also increase knowledge of the impact that the presence of a CD has on the emotional health of adolescents. Thus, it strengthens, the importance of undertaking group interventions with adolescents, ideally in the school environment, which enhance their self-esteem, emotional skills, and social support network. This type of program will help prevent or diminish emotional problems during this period. In addition, our research highlights the importance of considering the presence of CD currently in the life cycle.

In conclusion, knowing which variables are associated with the psychological health of adolescents can help reduce their impact on their physical and emotional well-being. It is necessary to promote emotional health in adolescents by increasing emotional skills, positive social relationships, and self-esteem, with special attention to adolescents with CD who need it.

## Data Availability Statement

The raw data supporting the conclusions of this article will be made available by the authors, without undue reservation, to any qualified researcher.

## Ethics Statement

The studies involving human participants were reviewed and approved by the Bioethics Committee of the Government of the Valencian Community (CN00A/2020/42/S). Written informed consent to participate in this study was provided by the participants’ legal guardian/next of kin.

## Author Contributions

All authors listed have made a substantial, direct and intellectual contribution to the work, and approved it for publication.

## Conflict of Interest

The authors declare that the research was conducted in the absence of any commercial or financial relationships that could be construed as a potential conflict of interest.
